# Incorporating High-Frequency Physiologic Data Using Computational Dictionary Learning Improves Prediction of Delayed Cerebral Ischemia Compared to Existing Methods

**DOI:** 10.3389/fneur.2018.00122

**Published:** 2018-03-07

**Authors:** Murad Megjhani, Kalijah Terilli, Hans-Peter Frey, Angela G. Velazquez, Kevin William Doyle, Edward Sander Connolly, David Jinou Roh, Sachin Agarwal, Jan Claassen, Noemie Elhadad, Soojin Park

**Affiliations:** ^1^Department of Neurology, Columbia University, New York, NY, United States; ^2^Department of Neurosurgery, Columbia University, New York, NY, United States; ^3^Department of Biomedical Informatics, Columbia University, New York, NY, United States

**Keywords:** subarachnoid hemorrhage, convolutional dictionary learning, time series, machine learning, critical care

## Abstract

**Purpose:**

Accurate prediction of delayed cerebral ischemia (DCI) after subarachnoid hemorrhage (SAH) can be critical for planning interventions to prevent poor neurological outcome. This paper presents a model using convolution dictionary learning to extract features from physiological data available from bedside monitors. We develop and validate a prediction model for DCI after SAH, demonstrating improved precision over standard methods alone.

**Methods:**

488 consecutive SAH admissions from 2006 to 2014 to a tertiary care hospital were included. Models were trained on 80%, while 20% were set aside for validation testing. Modified Fisher Scale was considered the standard grading scale in clinical use; baseline features also analyzed included age, sex, Hunt–Hess, and Glasgow Coma Scales. An unsupervised approach using convolution dictionary learning was used to extract features from physiological time series (systolic blood pressure and diastolic blood pressure, heart rate, respiratory rate, and oxygen saturation). Classifiers (partial least squares and linear and kernel support vector machines) were trained on feature subsets of the derivation dataset. Models were applied to the validation dataset.

**Results:**

The performances of the best classifiers on the validation dataset are reported by feature subset. Standard grading scale (mFS): AUC 0.54. Combined demographics and grading scales (baseline features): AUC 0.63. Kernel derived physiologic features: AUC 0.66. Combined baseline and physiologic features with redundant feature reduction: AUC 0.71 on derivation dataset and 0.78 on validation dataset.

**Conclusion:**

Current DCI prediction tools rely on admission imaging and are advantageously simple to employ. However, using an agnostic and computationally inexpensive learning approach for high-frequency physiologic time series data, we demonstrated that we could incorporate individual physiologic data to achieve higher classification accuracy.

## Introduction

Subarachnoid hemorrhage (SAH) is a major public health burden, affecting 14.5 per 100,000 persons in the United States alone (1, 2). Much of the resulting functional and cognitive disability is due to delayed cerebral ischemia (DCI) from vasospasm (VSP) (3–7). VSP refers to the narrowing of cerebral blood vessels triggered by the unusual presence of blood surrounding the vessel after a ruptured aneurysm, which can result in stroke. It occurs in 30% of SAH patients (8, 9) [54% of SAH patients in coma (10)]. DCI is a consensus definition with significance for normalizing research efforts in this disease and is defined as the development of new focal neurological signs or decrease of >2 points on the Glasgow Coma Scale (GCS), lasting for more than 1 h, or the appearance of new infarctions on CT or MRI (11, 12), excluding causes other than VSP.

As in other causes of stroke and secondary brain injury in the neurologic intensive care unit (NICU), time is of the essence to detect and intervene. Our interest is in predicting DCI and VSP with greater precision than standard of care scales that rely on admission assessments of blood patterns on computed tomography scans (13–16). For the higher risk SAH patients, the first 10–14 days are occupied by efforts to detect subtle examination changes that suggest VSP (17), and arrange urgent imaging to confirm VSP. For a syndrome with subtle symptoms and time sensitivity, it would be helpful to be more accurate in prediction so clinicians can focus resources, appropriately increase monitoring intensity, and justify diagnostic interventions to prevent permanent injury. On the converse, discharging patients from the ICU at low risk for DCI can result in significant cost savings (18).

Existing predictive models of DCI and VSP after spontaneous SAH are non-dynamic and while they help risk-stratify patients, they can lack accuracy and precision when applied to individuals (13–16). Efforts to improve this early prediction without additional monitoring have met moderate results, by combining risk scores (19), incorporating baseline features such as clinical condition and age (20), or assessment of autoregulation (21).

There is an abundance of physiologic and clinical data that are created and collected in the NICU. Few efforts have explored physiological data for the early prediction of DCI. In Ref. (22), a Naïve Bayes classifier using electronic medical record (EMR) data (cerebrospinal fluid drainage volume, sodium and glucose) and physiologic data [mean arterial blood pressure, heart rate (HR), and intracranial pressure] was able to classify patients for angiographic VSP with a moderately favorable AUC of 0.71. The raw data used in that study was low frequency (hourly at best) and extracted features summarized over 24 or 48 h. Despite the small sample size in that study, the result was encouraging that EMR and physiologic data could improve risk stratification for future events. The question remains whether increased precision can be achieved with use of higher frequency data. In this work, we applied machine-learning techniques, to extract features from the high-frequency data, to predict DCI. There is an extensive literature regarding robust feature extraction from physiological time series data for outcome prediction. Approaches can be broadly classified as either hypothesis driven or data driven. Hypothesis driven approaches have focused primarily on temporal data abstraction that relies on knowledge-based symbolic representations of clinical states, either by *a priori* threshold setting or interval changes (23, 24), summary statistics (22, 25–28), or template matching (29). Hypothesis driven feature extraction can be effective in prediction but requires domain expertise in designing meta-features and may introduce a bias (25).

Data driven or learning approaches such as used in this study extract meaningful features directly from the labeled data without *a priori* hypothesis (26, 27, 30–37). Sparse coding and dictionary learning methods (38–42) have shown promise in the field of image processing (38–40, 43–45) and have recently been applied to temporal data (46). Bahadori et al. (36) have used sparse clustering to extract the latent subspace for mortality prediction in the publicly available physionet ICU dataset. Lasko et al. (47) have extracted the temporal dynamics using auto encoders to identify the unlabeled phenotypes expressed in the sequences of serum uric acid signatures of gout vs acute leukemia. This work focuses on sparsity based data-driven techniques to extract the features to remain agnostic about scales, trends or patterns that might be available in the data as opposed to the hypothesis driven which summarizes the temporal data to extract features. In particular, we learned multiscale dictionaries from high-frequency temporal physiologic data that extract informative kernels that maximally classified for DCI.

## Materials and Methods

The proposed method is based on recent advances in convolution dictionary learning methods (38–40, 48). Convolution dictionary learning extract translation invariant kernels directly from the data, thus capturing the temporal characteristics of physiological variables. We extracted features using dictionaries learned from patients’ time series data acquired from bedside monitors. We learned multiscale dictionaries using convolution dictionary learning, by down sampling the data at increasing intervals (1, 5, 10, 20, 60, 120, and 240 min). This was intended to capture the temporal dynamics at different resolutions that might be available in the time-series data without *a priori* hypothesis. We learned the dictionaries for different physiological data variables using convolution dictionary learning as explained in the following sections.

Data analysis and model building were performed using custom software developed in Matlab 2016a (Mathworks, Natick, MA, USA) and Python (www.python.org). All computations were performed using an Intel Xeon CPU 2.2 GHz processor.

### Study Population

Consecutive patients with SAH admitted to the NICU between August 1996 and December 2014 were prospectively enrolled in an observational cohort study of SAH patients designed to identify novel risk factors for secondary injury and poor outcome. The study was approved by the Columbia University Medical Center Institutional Review Board. In all cases, written informed consent was obtained from the patient or a surrogate. SAH secondary to perimesencephalic bleeds, trauma, arteriovenous malformation, and patients <18 years old were not enrolled in the study. Starting in 2006, physiologic data was acquired using a high-resolution acquisition system (BedmasterEX; Excel Medical Electronics Inc., Jupiter, FL, USA) from General Electric Solar 8000i monitors (Port Washington, NY, USA; 2006–2013) or Philips Intellivue MP70 monitors (Amsterdam, The Netherlands; 2013–2014) at 0.2 Hz.

Exclusion criteria for this project were the following: (1) absence of physiologic monitoring data (before 2006), (2) VSP or DCI before post bleed day (PBD) 3, and (3) patients missing all candidate features. The targeted classification outcome was DCI, defined as development of new focal neurologic signs or deterioration of consciousness for >1 h or appearance of new infarctions on imaging due to VSP (12).

### Baseline Candidate Features

The following baseline characteristics and grading scales were prospectively recorded at admission: age, sex, worst Hunt–Hess grade in first 24 h (HH), mFS, and admission GCS. HH grade was dichotomized into low grade (1–3) and high grade (4–5). MFS was dichotomized into low grade (0–2) and high grade (3–4). Baseline features were compared for patients with DCI vs no DCI. Baseline features were also compared for the derivation vs validation dataset.

Frequency comparisons for categorical variables were performed by Fisher exact test. Two-group comparisons of continuous variables were performed with the Mann–Whitney *U* test. All statistical tests were two-tailed, and a *p*-value <0.05 was considered statistically significant.

### Physiological Data Extraction

Physiologic data was limited to the first 4 days after aneurysm rupture to limit the influence of clinical treatment in response to suspected VSP or DCI (17). While 0.2 Hz physiological data was available, we remained agnostic about the optimal scale or sampling rate for DCI classification. Five universally available ICU variables [HR, respiratory rate (RR), systolic blood pressure (SBP), diastolic blood pressure (DBP), and oxygen saturation (SPO_2_)] were downsampled (ds) from 0.2 Hz to 1, 5, 10, 20, 60, 120, and 240 min. Downsampling was computed as means, which also deals with erroneous or missing data (49). These variables were then used to learn the distinct temporal dynamics to derive features.

### Feature Extraction Using Convolution Dictionary Learning

Given a time series *X* ∈ *R*^1×^*^t^*, our method learns the distinct temporal dynamics by a dictionary based model for each of the five ICU variables. The term “dictionary” refers to the set of basis vectors that can be combined linearly to represent *X*. These basis vectors are learned directly from the data, and the size of the kernel must be big enough to capture the hidden patterns. In our work, we selected the kernels sizes 2, 5, 10, and 20 to learn multiscale dictionary as explained below.

#### Dictionary Learning Algorithm

Let *X* ∈ *R*^1×^*^t^* be the time series data. For a traditional dictionary learning algorithm, the time series data are then represented by set of patches {*x_i_*}*_i_*_=1,…,_*_N_*, where *x_i_* ∈ *R*^1×^*^r^*, which may be overlapping patches of size *r*. Given a set of patches, we learn an over-complete dictionary, denoted by *D* = {*d*_1_, …, *d_k_*} ∈ *R^r^*^×^*^K^* where *K* is the number of basis elements in dictionary *D*, usually referred to as “atoms” or “kernels.” The patch data are then approximated by *DГ*, where *Г* = {γ_1_, …, γ*_N_*} is a matrix of sparse vectors. The traditional dictionary learning algorithm then solves the following optimization:

$$\langle D,{\left\{\gamma \right\}}_{i}\rangle =\arg\underset{D,{\left\{\gamma \right\}}_{i}}{\min}\sum_{i=1}^{N}{\left\|x_{i}-D\gamma_{i}\right\|}_{2}^{2}+\lambda \sum_{i=1}^{N}{\left\|\gamma_{i}\right\|}_{1}.$$

The term ${\left\|x_{i}-D\gamma_{i}\right\|}_{2}^{2}$ denotes the signal reconstruction error. The reconstruction error is minimized subjected to *L*_1_ sparsity constraint on sparse vector γ*_i_*, where λ controls the sparsity. However, using a patch based dictionary results in redundant elements (12). Therefore, we chose to learn the convolution dictionary, which offers two main advantages, (i) the direct support for multiscale dictionaries and (ii) the patch size can be arbitrarily increased at negligible computation cost. The convolution dictionary algorithm learns kernels from the entire time series data instead of patches, thus resulting in fewer atoms. The convolution dictionary solves the following optimization:

$$\langle D,{\left\{\gamma \right\}}_{i}\rangle =\arg\underset{D,{\left\{\gamma \right\}}_{i}}{\min}{\left\|X-\sum_{k=1}^{K}d_{k}*\Gamma_{k}\right\|}_{2}^{2}+\lambda \sum_{K=1}^{K}{\left\|\Gamma_{k}\right\|}_{1},$$

where *Γ_k_* is the sparse activation map over the entire time series data and “*” denotes the convolution operation. By learning the convolution dictionary, we remove many redundant atoms that were simply shifted and clipped versions of the patches (38–40, 50).

To deal with missing/lost data beyond the scale of downsampling, we used mask decoupling (38–40) which introduces a masking operator to zero out missing data while minimizing the error. This requires solving the following optimization:

$$\langle D,\Gamma \rangle =\arg\underset{D,\Gamma }{\min}{\left\|X-WD\Gamma \right\|}_{2}^{2}+\lambda {\left\|\Gamma \right\|}_{1},$$

where *W* is a mask operator that zeros out any region with the missing data. The above optimization problem is solved using alternating direction method multipliers as described in Boyd et al. (51). Any other convolution dictionary learning method can be used to learn the atoms (52, 53). The dictionaries *D*, learned at different down sampling rates, are then used for deriving the features for DCI classification.

#### Feature Computation

Given a time series *X* ∈ *R*^1×^*^t^*, dictionary atom γ ∈ *R*^1×^*^k^*, where *k* ≤ *t*, the feature *f_i_* for series *X* and filter γ is given by max(X*γ), where * denotes the valid convolution. Valid convolution means that γ is applied only at each position of *X* such that γ lies within *X*. In other words, we performed convolutions only when the contiguous data length was twice the length of kernel. The number of kernels that we extracted from the data was discovered through an optimization step to find maximal model performance. We therefore extracted 20 kernels for each varying kernel length (KL; 2, 5, 8, 10, 20, and 40) and for each downsampling period (ds; 1, 5, 10, 20, 60, 120, and 240 min), and for each of five variables (var; HR, RR, SBP, DBP, and SPO_2_). This resulted in 4,200 candidate kernel derived physiological features.

#### Feature Selection and Model Building

We used the above features to develop models using three different classifiers (partial least square, SVM linear, and SVM kernel). Minimal redundancy maximal relevance (mRMR) (54) was applied to identify the most relevant features for classification. mRMR selects the features that maximize the mutual information between features and target class, and minimizes mutual information among the features. The features are ranked based on the greedy search that maximizes the Mutual Information Difference Criterion or Mutual Information Quotient Criterion. Let *S* ∈ (*x*_1_, …, *x_n_*) be the set of features and *h* be the target class (in our case DCI vs non-DCI), then the features are ranked as follows:

$$\text{MID}:\underset{x_{i}\in S}{\max}\left[I(x_{i},h)-\frac{1}{\left|S\right|}\underset{x_{j}\in S}{\sum }I(x_{i},x_{j})\right],$$

$$\text{MIQ}:\underset{x_{i}\in S}{\max}\left[\frac{I(x_{i},h)}{\frac{1}{\left|S\right|}\sum_{x_{j}\in S}I(x_{i},x_{j})}\right],$$

where, *I*(*x_i_, h*) is the information gain between the feature *x_i_* and target class *h*. The first “*k*” ranked features are then used to learn the classifier. This simplifies the model, reduces training times, and enhances the generalizability of the classification model.

We used mRMR in combination with linear and kernel based support vector machines (SVM-L and SMV-K) classifiers (55, 56), as well as partial least squares (PLS) regression (57) for combined feature selection and classification. PLS regression performs a principal component analysis on all feature vectors first and then applies a least squares regression using those components that explain the most variance. Weighted SVM (58) was utilized to account for the imbalance in classification categories (i.e., fewer DCI vs non-DCI in any consecutive SAH dataset). We created models using baseline features, physiological features and combined baseline and physiological features, along with feature selection to test the discriminative ability of different features. We compared the performance of the physiological features learned from our model with the baseline and grading scale features.

### Internal Validation and Validation Strategy

**Figure 1:**
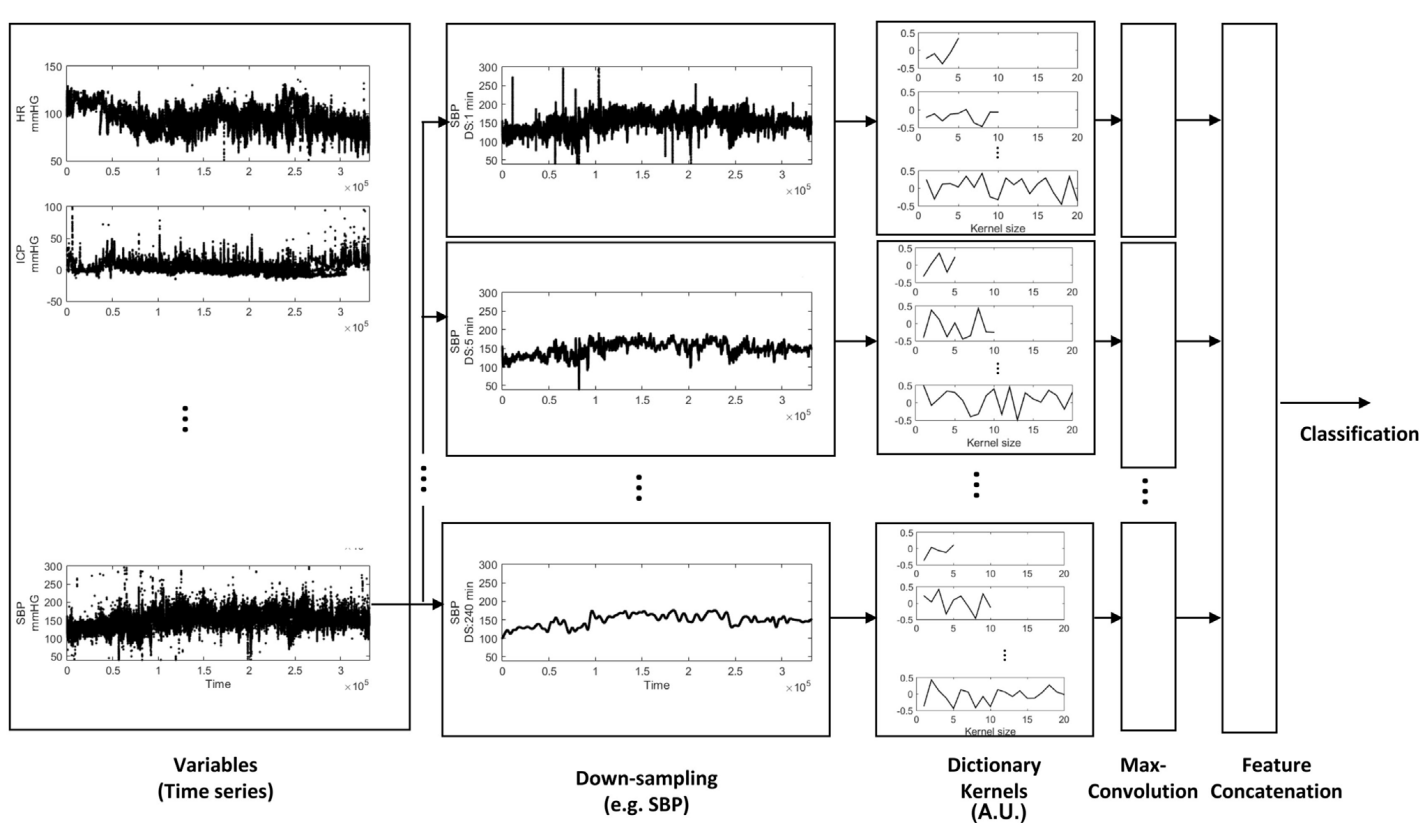
Feature extraction from physiologic time series data. Time-series variables were downsampled, and the dictionary was learned to extract different temporal patterns presented at different down sampling rates. The dictionary kernels presented here capture the temporal dynamics (extracted features) for classification of delayed cerebral ischemia.

The cohort was randomly split 80/20%, while maintaining proportional targeted outcome (DCI). 80% were used to learn the dictionary and train/test models, and considered the primary derivation dataset. For internal validation of our models, we performed cross-validation of the derivation data with a 12.5% hold-out set; the hold-out set was proportional to the training data set for percentage of targeted outcome. Discriminative performance is described by an area under the receiver operating characteristic curve (AUC), and AUCs were statistically compared (59). The median value of AUC is reported, over 100 runs. 20% of the cohort were not involved in model training, and used exclusively for testing the classification accuracy of our models. Classification accuracy of our models on the validation test set is reported as AUC, with 95% confidence intervals (CI). An overview of the analytical approach is illustrated in Figure 1. To summarize, physiological variables were downsampled to extract temporal patterns at varying scales using dictionary learning. We extracted 20 kernels for each varying KL (2, 5, 8, 10, 20, and 40) and for each downsampling period (ds; 1, 5, 10, 20, 60, 120, and 240 min), and for each of five variables (var; HR, RR, SBP, DBP, and SPO_2_). The resulting kernels were convolved with time series at different scales to extract the maximal value resulting in 4,200 features; the dimensions of these features were reduced by mRMR to classify DCI using PLS, SVM-L, and SVM-K.

## Results

From May 2006 to December 2014, 562 SAH patients with physiologic data were enrolled. 8 had VSP or DCI identified before PBD 3, 66 were missing all candidate features leaving a total of 488 subjects included in the study. The median AUC of 100 runs of cross-validation (with 12.5% hold-out set) is presented in Table 1.

**Table 1:** Model performance in derivation and validation datasets for, partial least squares (PLS), support vector machines linear and kernel (SVM-L and SVM-K).

Features	Derivation dataset (median AUC of 100 runs)			Validation dataset [AUC (95% confidence intervals)]		
	Classifiers			Classifiers		
	PLS	SVM-L	SVM-K	PLS	SVM-L	SVM-K
Age	0.58	0.54	0.53	0.58 (0.46–0.7)	0.6 (0.48–0.71)	0.64 (0.53–0.76)
Sex	0.59	0.59	0.59	0.62 (0.5–0.74)	0.62 (0.5–0.74)	0.62 (0.5–0.74)
Hunt Hess Scale	0.60	0.55	0.58	0.49 (0.37–0.61)	0.46 (0.34–0.58)	0.5 (0.38–0.62)
Modified Fisher Scale	0.54	0.57	0.50	0.47 (0.35–0.59)	0.53 (0.41–0.65)	0.53 (0.41–0.65)
Glasgow Coma Scale	0.59	0.57	0.63	0.43 (0.31–0.55)	0.44 (0.32–0.56)	0.56 (0.44–0.68)
Baseline (age, sex, and scales)	0.63	0.58	0.54	0.64 (0.53–0.76)	0.59 (0.48–0.71)	0.61 (0.49–0.72)
Diastolic blood pressure	0.52	0.48	0.56	0.44 (0.26–0.61)	0.42 (0.25–0.59)	0.56 (0.19–0.53)
Systolic blood pressure	0.58	0.54	0.49	0.65 (0.49–0.82)	0.43 (0.26–0.6)	0.36 (0.19–0.53)
Heart rate	0.55	0.51	0.50	0.46 (0.28–0.63)	0.5 (0.33–0.68)	0.45 (0.28–0.62)
Oxygen saturation	0.56	0.53	0.50	0.62 (0.45–0.79)	0.48 (0.31–0.65)	0.5 (0.33–0.67)
Respiratory rate	0.49	0.50	0.50	0.57 (0.4–0.74)	0.54 (0.36–0.71)	0.5 (0.33–0.67)
Combined physiological	0.66	0.56	0.50	0.47 (0.3–0.64)	0.51 (0.34–0.68)	0.5 (0.33–0.67)
Baseline and physiological	0.63	0.56	0.50	0.5 (0.33–0.67)	0.5 (0.33–0.67)	0.5 (0.33–0.67)
MRMR (baseline and physiological)	**0.71**	0.60	0.50	**0.78** (0.64–0.92)	0.64 (0.47–0.8)	0.5 (0.33–0.67)

*The SVM-L classifier with maximal relevance and minimal redundancy (MRMR) feature reduction performed the best*.

*Values highlighted in bold indicates the performance of the classifier that performed the best*.

### Baseline Feature Model Performance

Among demographical information, sex (AUC 0.59) performed slightly better than age (AUC 0.58, PLS). GCS (AUC 0.63, SVM-K) achieved slightly better accuracy than HH (AUC 0.60, PLS) and MFS (AUC 0.57, SVM-L). By combining demographics and grading scales (age, sex, HH, mFS, and GCS), a PLS classifier performed better than the individual features with an AUC of 0.63 in predicting DCI.

The classification accuracy on the validation set was found to be similar to the derivation set. A model based on current standard grading scale (MFS) achieved an AUC of 0.56 (SVM-L, 95% CI, 0.44–0.67). Combining all the demographics and grading scales improved the AUC to 0.64 (PLS, 95% CI, 0.52–0.76).

### Physiological Feature Model Performance

Features extracted from individual physiological time-series variables did not perform significantly better than the baseline features. SBP (AUC 0.58, PLS) achieved slightly better AUC than the other variables. However, adding all the features derived from physiological data achieved an AUC of 0.66. Adding demographics and grading scales along with the feature reduction performed better than individual features by achieving an AUC of 0.71, which was statistically significantly higher than the performance of MFS (AUC of 0.57, *p* = 0.0025).

The performance on the validation set was found to be similar to that of the derivation set. Feature reduction (to reduce redundancy and maximal relevance) when applied to combined demographics, grading scales, and physiological data produced the best classification performance with an AUC of 0.78 (PLS, 95% CI, 0.63–0.92).

**Figure 2:**
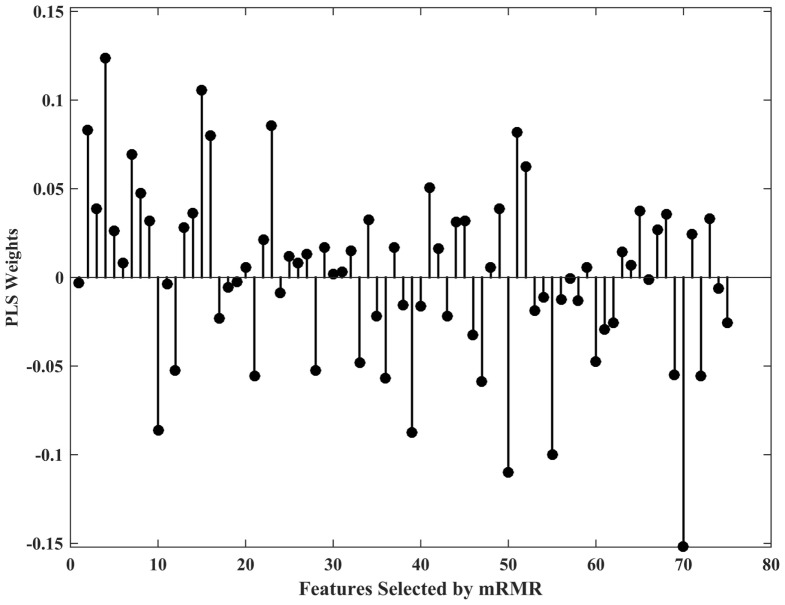
Partial least squares (PLS) classifier weights of 80 features.

**Figure 3:**
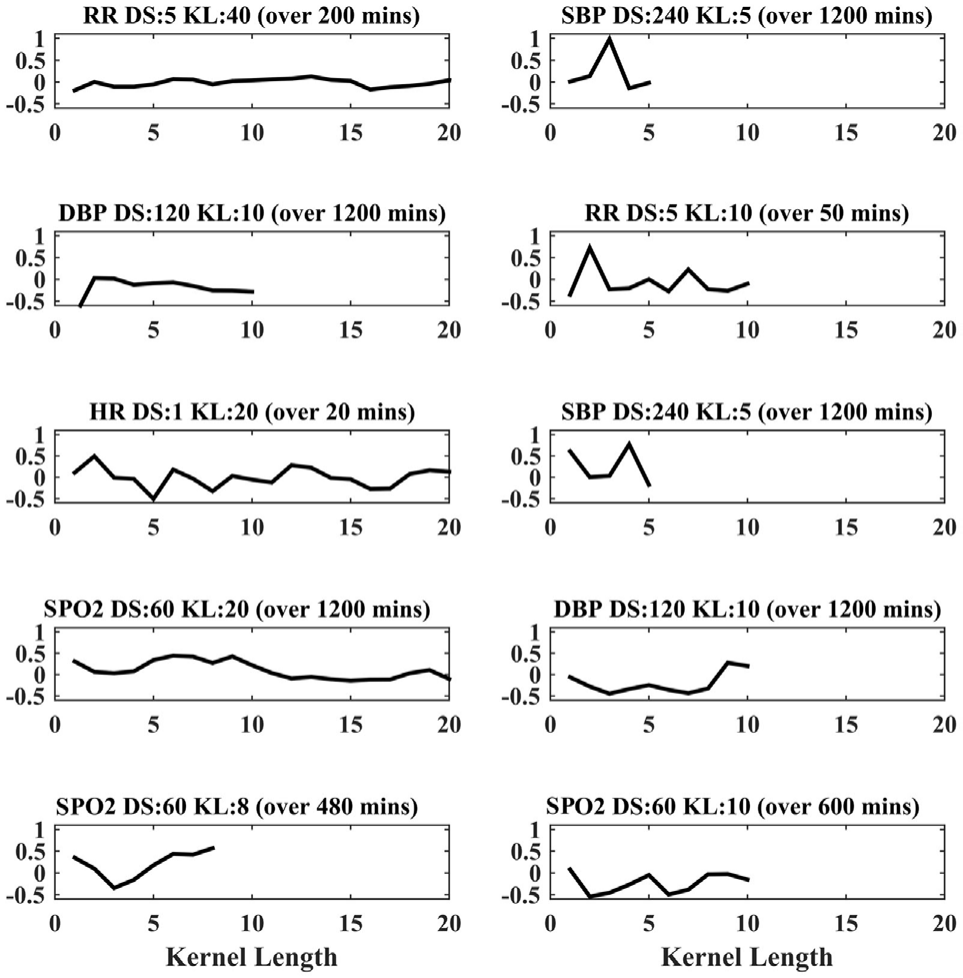
Feature extraction from physiological time-series data. Top 10 representative kernels are demonstrated for varying kernel length (KL), down sampling rate (DS) highlighting the need for 20 kernels were extracted for maximal convolution, for each varying (KL; 2, 5, 10, and 20) and for each downsampling period (ds; 1, 5, 10, 20, 60, 120, and 240 min), and for each of respiratory rate (RR), top 10 kernels.

In the case of the PLS classifier, the weights indicate the discriminative power of the features in separating the two classes. Figure 2 shows the PLS weights of the 80 features. Figure 3 shows the kernels corresponding to a demonstrative selection of the top 10 features relevant for classification. The kernel displays the time varying characteristics for different variables and highlights the need for capturing high-frequency data at different scales (downsampling rate).

## Discussion

Recognizing trends and patterns, and minutely analyzing complex data requires the layered knowledge of clinical experts, but defies rule-based systems. It has previously been shown that a Naïve Bayes classifier using summary statistics of 24 h (low frequency) data can classify for angiographic VSP better than clinician-dependent Dopplers and exams (22). Here, we show that features extracted from higher frequency temporal data (ranging from 1 min to 4 h) is superior to gold standard grading scales in classification of DCI after SAH. In our approach, we extracted high-level features from existing physiologic data, without an *a priori* hypothesis of what patterns might emerge. There are two parts to our algorithm; first is extracting kernels from the data using convolution dictionary learning, which is computationally expensive but can be performed offline and once (kernel generation for five variables for six KLs and for seven downsampling rates took 1 day). Second is the model building, this is relatively faster and is on the order of minutes; this, too, can be performed offline and once. Once the model is built, it can be applied for each patient in a clinical setting on day 4 of SAH, with a computational time on the order of seconds. Our approach is more accurate than a gold standard grading scale and viable in a clinical setting.

To enable validation efforts and generalizability to other datasets and institutions, we focused on universal physiologic ICU variables and typical baseline grading scales pertinent to SAH used in the NICU. In this translational work, we used a dictionary learning method to extract frequency selective and translation invariant characteristics of time series data. To the best of our knowledge, this is the first study that shows the efficacy of dictionary learning for DCI classification using time series. The novel application to time series data required some choices bound by characteristics of the dataset (KLs) and domain (downsampling rates). We tested our method for its discriminative ability for DCI and found that dictionary kernel derived physiological features outperformed a gold standard static grading scale. When combined with grading scales and demographics, our dictionary learning based method predicted DCI with an AUC (0.71, PLS) approaching clinical reliability [threshold of 0.8 (60)]. A model with MFS alone had an AUC of 0.57. While a comparison of AUCs is frequently used in biostatistics and computer science to demonstrate trends of improvement between tools, it is not sensitive enough to accurately capture improvements in predictive discrimination (61). We therefore compared the AUCs using Hanley’s method of comparing ROCs of specific tools in the same population, which showed the statistical significance of the difference between the two models’ ROCs (59).

An effort to show robustness of the model was with an internal validation strategy, testing on a separate dataset excluded from model building entirely. Generalizability of a machine-learning algorithm, however, assumes that the training dataset is large and diverse enough to be representative. A limitation of our study is the possibility of causality leakage (62). We attempted to limit effects of causality leakage, i.e., the influence of cerebral perfusion efforts on our data, by censoring beyond day 4, which is the highest risk of onset of DCI. Another limitation to this study is the single center approach; there is no publicly available dataset for SAH with similar granularity of physiologic data. Future efforts will include developing complementary SAH cohorts and validating these algorithms on other centers’ data.

## Conclusion

A data-driven dictionary based featurization and learning approach to physiological time series data prior to peak DCI period shows promise to improve prediction precision. This is a computationally inexpensive and agnostic feature extraction approach for physiologic time series parameters in the ICU (HR, RR, SBP, DBP, and SPO_2_). There is a vast pool of candidate features within the EMR with a biological basis for classification ability (i.e., drawn from frequentist statistical studies showing relationship with VSP and DCI in specific SAH cohorts). Future efforts will also draw from this feature pool to further improve the precision of DCI prediction, favoring those candidate features that are obtained for standard clinical care and thus potentially automatable.

## Ethics Statement

Consecutive patients with aneurysmal SAH admitted to the NICU between August 1996 and December 2014 were prospectively enrolled in an observational cohort study of SAH patients designed to identify novel risk factors for secondary injury and poor outcome. The study was approved by the Columbia University Medical Center Institutional Review Board. In all cases, written informed consent was obtained from the patient or a surrogate.

## Author Contributions

SP, JC, SA, DR, EC, AV, KD and HF: Data collection. MM, SP, and KT: Analysis. MM and SP: writing. MM, KT, HF, AV, KD, EC, DR, SA, JC, NE and SP: Editing.

## Conflict of Interest Statement

The authors declare that the research was conducted in the absence of any commercial or financial relationships that could be construed as a potential conflict of interest. The reviewers LC and IS and handling editor declared their shared affiliation.
